# Quantitative comparison of data-driven gating and external hardware gating for ^18^F-FDG PET-MRI in patients with esophageal tumors

**DOI:** 10.1186/s41824-021-00099-x

**Published:** 2021-03-23

**Authors:** Sofia Kvernby, Nafsika Korsavidou Hult, Elin Lindström, Jonathan Sigfridsson, Gustav Linder, Jakob Hedberg, Håkan Ahlström, Tomas Bjerner, Mark Lubberink

**Affiliations:** 1 Radiology & Nuclear Medicine, Department of Surgical Sciences, Uppsala University, Uppsala, Sweden; 2 Medical Physics, Uppsala University Hospital, Entrance 85, SE-751 85 Uppsala, Sweden; 3 Medical Imaging Centre, Uppsala University Hospital, Entrance 85, SE-751 85 Uppsala, Sweden; 4 Section of Gastrointestinal Surgery, Department of Surgical Sciences, Uppsala University, Uppsala, Sweden; 5 Antaros Medical AB, Mölndal, Sweden

**Keywords:** Respiratory gating, DDG, PET-MRI, Esophageal tumors

## Abstract

**Background:**

Respiratory motion during PET imaging reduces image quality. Data-driven gating (DDG) based on principal component analysis (PCA) can be used to identify respiratory signals. The use of DDG, without need for external devices, would greatly increase the feasibility of using respiratory gating in a routine clinical setting. The objective of this study was to evaluate data-driven gating in relation to external hardware gating and regular static image acquisition on PET-MRI data with respect to SUV_max_ and lesion volumes.

**Methods:**

Sixteen patients with esophageal or gastroesophageal cancer (Siewert I and II) underwent a 6-min PET scan on a Signa PET-MRI system (GE Healthcare) 1.5–2 h after injection of 4 MBq/kg ^18^F-FDG. External hardware gating was done using a respiratory bellow device, and DDG was performed using MotionFree (GE Healthcare). The DDG raw data files and the external hardware-gating raw files were created on a Matlab-based toolbox from the whole 6-min scan LIST-file. For comparison, two 3-min static raw files were created for each patient. Images were reconstructed using TF-OSEM with resolution recovery with 2 iterations, 28 subsets, and 3-mm post filter. SUV_max_ and lesion volume were measured in all visible lesions, and noise level was measured in the liver. Paired *t*-test, linear regression, Pearson correlation, and Bland-Altman analysis were used to investigate difference, correlation, and agreement between the methods.

**Results:**

A total number of 30 lesions were included in the study. No significant differences between DDG and external hardware-gating SUV_max_ or lesion volumes were found, but the noise level was significantly reduced in the DDG images. Both DDG and external hardware gating demonstrated significantly higher SUV_max_ (9.4% for DDG, 10.3% for external hardware gating) and smaller lesion volume (− 5.4% for DDG, − 6.6% for external gating) in comparison with non-gated static images.

**Conclusions:**

Data-driven gating with MotionFree for PET-MRI performed similar to external device gating for esophageal lesions with respect to SUV_max_ and lesion volume. Both gating methods significantly increased the SUV_max_ and reduced the lesion volume in comparison with non-gated static acquisition. DDG resulted in reduced image noise compared to external device gating and static images.

## Background

Respiratory motion during PET imaging of anatomical structures in the thorax and upper abdomen reduces image quality, resulting in decreased lesion detection and quantitative accuracy by introducing an underestimation of the standardized uptake value (SUV) and an overestimation of the lesion volume (Liu et al. 2009). Correction for respiratory motion in PET images can be performed in various ways. External hardware gating is based on tracking breathing motion externally, either by visually recording the movement of the chest wall or by using an external bellow device and then splitting the breathing cycle into different phases. Image reconstruction can then be performed by only using data from static gates of the breathing cycle, usually during exhale position (quiescent phase gating). To avoid decreased signal-to-noise ratio (SNR) due to reduction in count statistics, this method requires prolonged acquisition times.

For PET/MRI scanners, different MRI techniques can be used to detect motion. For example, analysis of shifts and rotations in k-space data can be used to estimate motion (Grimm et al. 2015), MRI image tagging can be used to detect deformation of tissue (Guérin et al. 2011), and very fast imaging sequences and navigator techniques can be used for tracing respiratory motion during PET-acquisition (Würslin et al. 2013), but in that case at the expense of reduced time to acquire clinically relevant MRI sequences and putting constraints on the PET/MRI imaging protocol.

Principal component analysis (PCA), which is a mathematical technique investigating changes in a dataset, can be used to extract respiratory motion patterns from raw PET list-mode data. Data-driven gating (DDG) based on PCA can be used to identify respiratory signals, similarly as external hardware gating does, and sort data into respiratory phases containing no, or very little, respiratory motion. DDG was first introduced by Thielemans et al. in 2011 (Thielemans et al. 2011) and has demonstrated promising results in several studies on PET-CT systems (Büther et al. 2016; Walker et al. 2019), showing high agreement with external device gating. On PET/MRI systems, device-less DDG has been used in combination with a short additional dynamic MRI scan for correction of breathing motion with good results (Manber et al. 2015).

Recently, GE Healthcare released a version of DDG based on PCA for PET-MRI scanners, as part of a research agreement, implemented in a Matlab-based Toolbox (Duetto, GE Healthcare, Waukesha). The use of DDG, without need for external devices, would greatly increase the feasibility of using respiratory gating in a routine clinical setting. The objective of this study is to evaluate DDG in relation to external hardware gating and regular static image acquisition on PET-MRI data in patients with esophageal cancer.

## Methods

Sixteen patients with esophageal and gastroesophageal junctional (GEJ) tumors were included in the study. Patient characteristics are described in detail in another study (Linder et al. 2019); in brief, inclusion criteria were potentially resectable tumors (T_1-4a_, N_1-3_, M_0_) and histologically verified esophageal or GEJ cancer (Siewert I and II). The patients were asked for study participation in the clinical setting after cancer diagnosis. PET-MRI was performed in conjunction with the routine PET-CT investigation to conclude clinical staging. All patients provided written informed consent to participate in the study, and approval was granted from the regional medical ethics committee in Uppsala (DNR 2014/551).

### Image acquisition and reconstruction

All patients underwent a 6-min PET scan on a 3T Signa PET-MRI system (GE Healthcare) 109 ± 21 min after injection of 4 MBq/kg ^18^F-FDG. The PET system has an axial and transaxial field of view (FOV) of 25 cm and 60 cm, respectively, producing 89 image planes with a slice thickness of 2.8 mm. Data were acquired in list mode to enable reconstruction of data in different time frames and breathing gates retrospectively.

External hardware gating was performed using an MRI-compatible respiratory bellow device, and a quiescent phase-based gating method (Q.Static, GE Healthcare) was used for the whole 6-min scan. Static gates were automatically extracted during end-expiration of the breathing cycle to form a gating phase free from motion (Soussan et al. 2016). The external gating triggers were stored in the PET list file, which enables retrospective unlisting of gated data during the quiescent phase. The quiescent phase includes 50% of the total breathing cycle, resulting in maintained PET coincidence data equivalent to a 3-min static scan.

The DDG was performed using MotionFree (GE Healthcare), which uses PCA to derive the respiratory waveforms directly from the PET coincidence data. In short, data are binned into 0.5-s sinograms, and the frequency of the motion can be determined by using the Fourier transform. Respiratory motion is defined as motion originating from frequencies in the range of 0.1–0.4 Hz, implying a respiratory cycle of 2.5–10 s. To establish the impact of the motion on the data, an *R* value is calculated as the ratio between the peak value within this respiratory frequency range and the mean value above the respiratory frequency range.

The whole 6-min PET list file was transferred to a Matlab-based toolbox containing the DDG MotionFree package (Duetto v02.03, GE Healthcare). In the toolbox, respiratory waveforms are derived from the PET-coincidence data, and DDG respiratory gating triggers are stored in the PET list file in the same way as for the external gating triggers. For the data-driven gating, an *R* value was given for each patient, describing the impact of respiratory motion on the data, where a threshold of *R*=15 is the default value recommendation for MotionFree.

Raw files were created in the toolbox by unlisting the static gates from the whole 6-min scan list file. For comparison with the gated data and for investigation of normal image quality variation in a scan-rescan situation, two 3-min static raw files (0–3 min and 3–6 min) were also created for each patient.

All images were reconstructed using TF-OSEM with resolution recovery, 2 iterations, 28 subsets, and a 3-mm Gaussian post-processing filter. A 60-cm FOV was used with a 192 × 192 reconstruction matrix, resulting in a 3.125 × 3.125 × 2.80 mm^3^ voxel size.

### Image analysis

SUV images were calculated by dividing the activity concentrations with the amount of injected activity per body weight. Images were visually assessed with respect to image artifacts and reconstruction errors. Esophageal lesions and lymph nodes were identified on the PET images, and SUV_max_ as well as lesion volume, based on a 41% SUV_max_ threshold (Boellaard et al. 2015), were measured using Hermes Affinity Viewer 1.1 (Hermes Medical Solutions). For image noise measurement, a spherical volume of interest with a diameter of 3 cm was placed in the liver in all images, and SUV mean values together with standard deviations were measured. Noise level was defined as the standard deviation divided by the mean SUV. To estimate the effect of lesion position on the efficacy of DDG, the vertical distance from the center of the lesion to the top of the liver was measured. Lesions below 1 cm^3^ were represented separately in graphs.

### Statistics

Results are presented as mean ± standard deviation unless otherwise specified. Data representing SUV_max_ and lesion volume were not normally distributed, while data representing changes in these parameters were normally distributed according to Shapiro-Wilk normality tests. To determine whether SUV_max_ and lesion volume measured in images with either of the two respiratory motion correction methods, external hardware gating and DDG, differed from those of a static non motion-corrected image, a non-parametric paired Wilcoxon test was used. Correlation between both gating methods and non-gated images was assessed using linear regression and Spearman correlation coefficients for SUV_max_ and lesion volume. To investigate agreement between DDG and external hardware gating, Bland-Altman analysis was performed for SUV_max_ and lesion volume values. Correlation between the distance from the lesion to the diaphragm, and the increase in SUV_max_ respectively the decrease in lesion volume, were assessed using Pearson correlation coefficients. Similarly, the correlation between the DDG *R* value, and the increase in SUV_max_ respectively the decrease in lesion volume, were assessed using Pearson correlation coefficients.

## Results

Sixteen PET-MRI examinations and a total number of 30 lesions (up to four lesions and lymph nodes per subject) were included in the study. The average *R* value given by DDG was 17.9 (range 6.9–28.7). In 75% of the patients, the DDG analysis estimated an *R* value greater than 15, implying a significant impact from breathing motion. Hence, only patients with an *R* value > 15 were included in the further analysis, in total 12 patients and 23 lesions.

In DDG images, the average lesion volume and SUV_max_ were 6.3 ± 9.1 cm^3^ (mean ± SD; range 0.14–32.0 cm^3^) and 30.8 ± 27.4 g/ml (range 8.8–130.6 g/ml). For external device-gated images, the average lesion volume and SUV_max_ were 6.2 ± 9.0 cm^3^ (mean ± SD; range 0.19–31.2 cm^3^) and 31.0 ± 28.4 g/ml (range 9.3–134.8 g/ml).

**Fig. 1:**
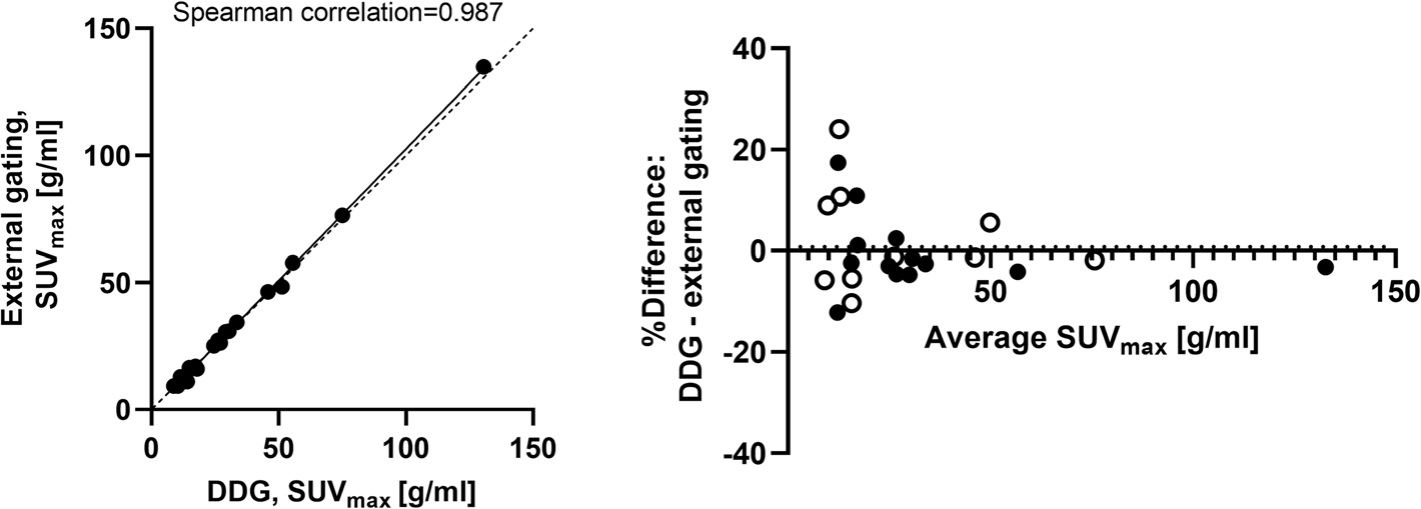
SUV_max_, correlation (left) and Bland-Altman analysis (right) between DDG and external device gating. Lesions with a volume < 1 cm^3^ are illustrated by unfilled circles in the Bland-Altman analysis. Dotted line represents line of identity (left graph) respectively bias (right graph)

**Fig. 2:**
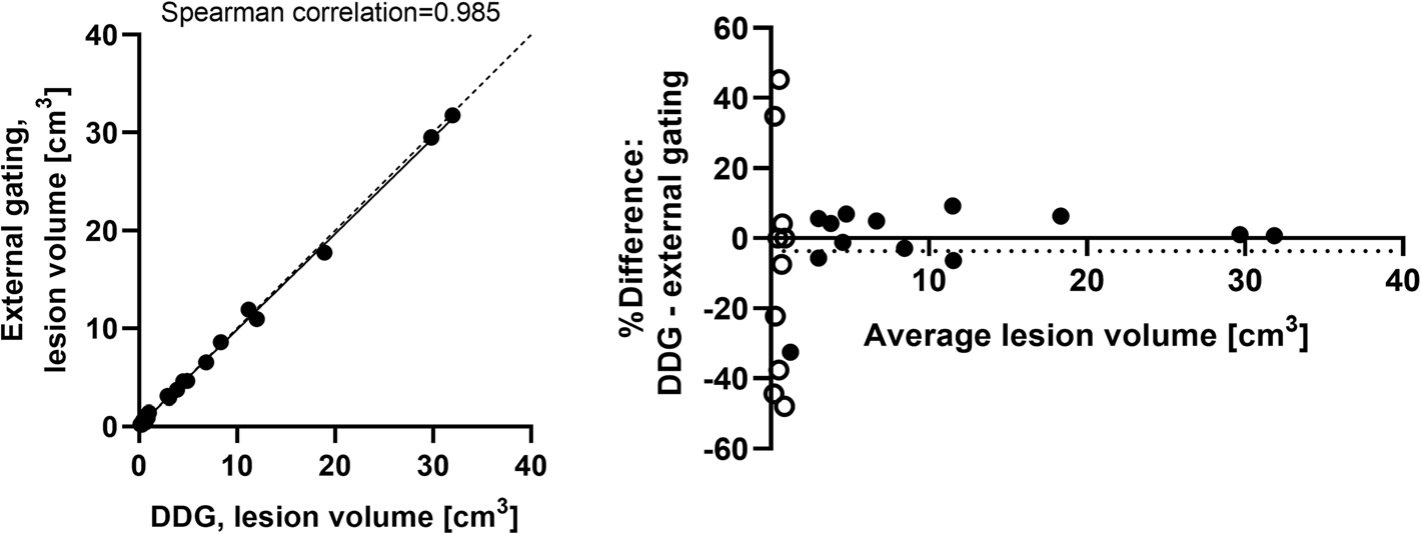
Lesion volume, correlation (left) and Bland-Altman analysis (right) between DDG and external device gating. Lesions with a volume < 1 cm^3^ are illustrated by unfilled circles in the Bland-Altman analysis. Dotted line represents line of identity (left graph) respectively bias (right graph)

No significant differences (*p*>0.05) were found between the data-driven gating and the external hardware-gating SUV_max_ or lesion volumes; a good correlation (*R*^2^=0.987 for SUV_max_ and *R*^2^=0.985 for lesion volume) between the methods could be seen. A Bland-Altman analysis was performed to compare and visually illustrate data from the two gating methods, which demonstrated a small non-significant bias (0.7% for SUV_max_ and −3.7% for lesion volume) with 95% limits of agreement of −16.2 to 17.7% for SUV_max_ and −46.7 to 39.3% for lesion volume (Figs. 1 and 2). Excluding lesions < 1cm^3^, as indicated by the open symbols in Figs. 1 and 2, showed even higher agreement between DDG and external gating (bias −0.5% for SUV_max_ and 0.2% for lesion volume; the 95% limits of agreement were −15.1 to 14.1% for SUV_max_ and −0.9 to 1.2% for lesion volume).

In static non-gated images, the average lesion volume and SUV_max_ were 6.3 ± 9.2 cm^3^ (mean ± SD; range 0.2–31.3 cm^3^) and 30.7 ± 23.8 g/ml (range 10.3–119.5 g/ml) for the first 3 min of acquisition and 6.7 ± 9.2 cm^3^ (range 0.3–31.4 cm^3^) and 28.1 ± 22.8 g/ml (range 6.9–112.8 g/ml) for the last 3 min of acquisition.

**Fig. 3:**
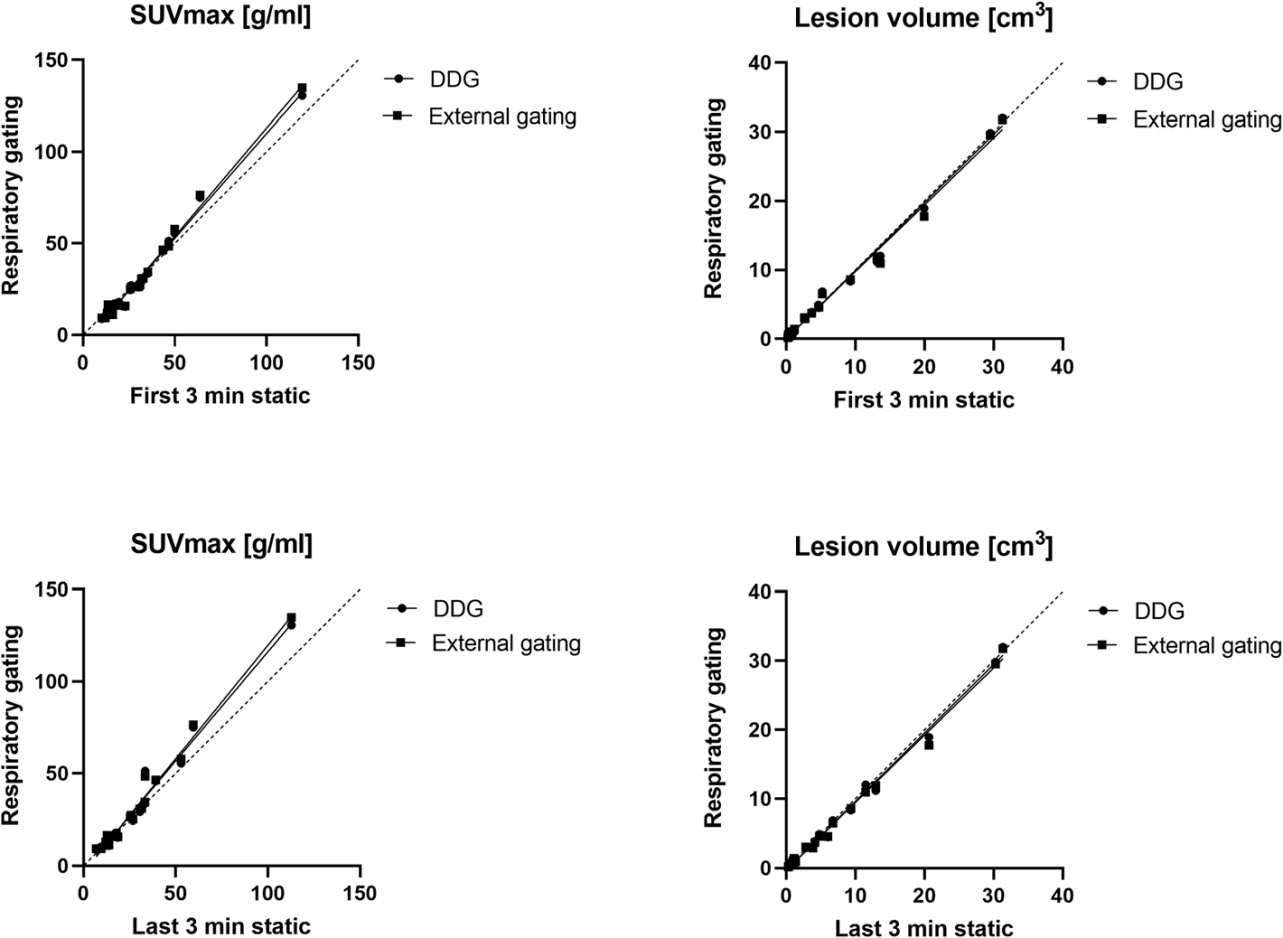
Correlation plots between gating methods and regular static acquisition for SUV_max_ (left) and lesion volumes based on a 41% SUV_max_ threshold (right). Upper graphs: correlation against static acquisition based on the first 3 min of data acquisition. Lower graphs: correlation against static acquisition based on the last 3 min of data acquisition

Both external device gating and DDG significantly influenced SUV_max_ and lesion volumes compared with the static non-gated images. The average SUV_max_ increased significantly (*p*<0.05) compared with the static acquisition based on data from the last 3 min of acquisition, for both DDG (9.4% increase, from 28.1 to 30.8) and the external hardware-gating method (10.3% increase, from 28.1 to 31.0). Furthermore, the average lesion volume decreased significantly (*p*<0.05) for both DDG (−5.4% decrease, from 6.7 to 6.3 cm^3^) and external device gating (−6.6% from 6.7 to 6.2 cm^3^). When instead comparing the gated data with the first 3 min of static acquisition, similar improvement in SUV_max_ and lesion volumes could be found, but this was not significant (*p*>0.05). Results are illustrated in Fig. 3.

Evaluating the correlation between the lesion’s distance to the diaphragm and the improvement in image accuracy (increase in SUV_max_ or decrease in lesion volume), the results did not demonstrate any clear correlation between respiratory gating methods and a static acquisition (Fig. 5). Similarly, the results did not demonstrate any strong correlation between *R* value and increase in SUV_max_ or decrease in lesion volume (Fig. 6).

Quantitative measures of noise level in the liver are presented in Table 1. The results demonstrated that the noise level in images based on data-driven gating, 0.165, was significantly lower (*p*<0.001) than in images based on either external hardware gating (noise level 0.235) or regular static acquisition (noise level 0.223 and 0.225).

**Table 1:** Quantitative measures in a liver sphere with a diameter of 3 cm. Data represents mean value and range (* indicates significantly different value, *p*<0.01, for DDG compared with either static or external device-gated acquisitions)

	Static acquisition		Gated acquisition	
Measure	First 0–3 min	Last 4–6 min	External device	DDG
SUV_mean_	2.37 (1.59–2.73)	2.31 (1.62–2.67)	2.27 (1.54–2.65)	2.27 (1.56–2.63)
SUV_SD_	0.53 (0.37–0.83)	0.52 (0.37–0.74)	0.53 (0.35–0.76)	0.37 (0.26–0.61)*
Noise level	0.225 (0.15–0.33)	0.223 (0.15–0.29)	0.235 (0.15–0.32)	0.165 (0.12–0.21)*
SNR	4.61 (3.07–6.51)	4.73 (3.05–6.45)	4.46 (2.96–6.46)	6.32 (4.67–8.50)*

## Discussion

In this study, we evaluated data-driven gating with MotionFree against external hardware gating and static acquisition. We found that data-driven gating performs equivalent to external hardware gating for esophageal lesions in a PET-MRI scanner with respect to lesion volume and SUV_max_. Our findings are in accordance with the results from recently published studies using DDG performed on PET-CT systems (Walker et al. 2018; Büther et al. 2020; Bertolli et al. 2018).

Both data-driven gating and external hardware gating retained ~50% of the coincidence data from a 6-min scan and significantly increased SUV_max_ and decreased lesion volume compared with a static 3-min acquisition that maintained all coincidence data.

**Fig. 4:**
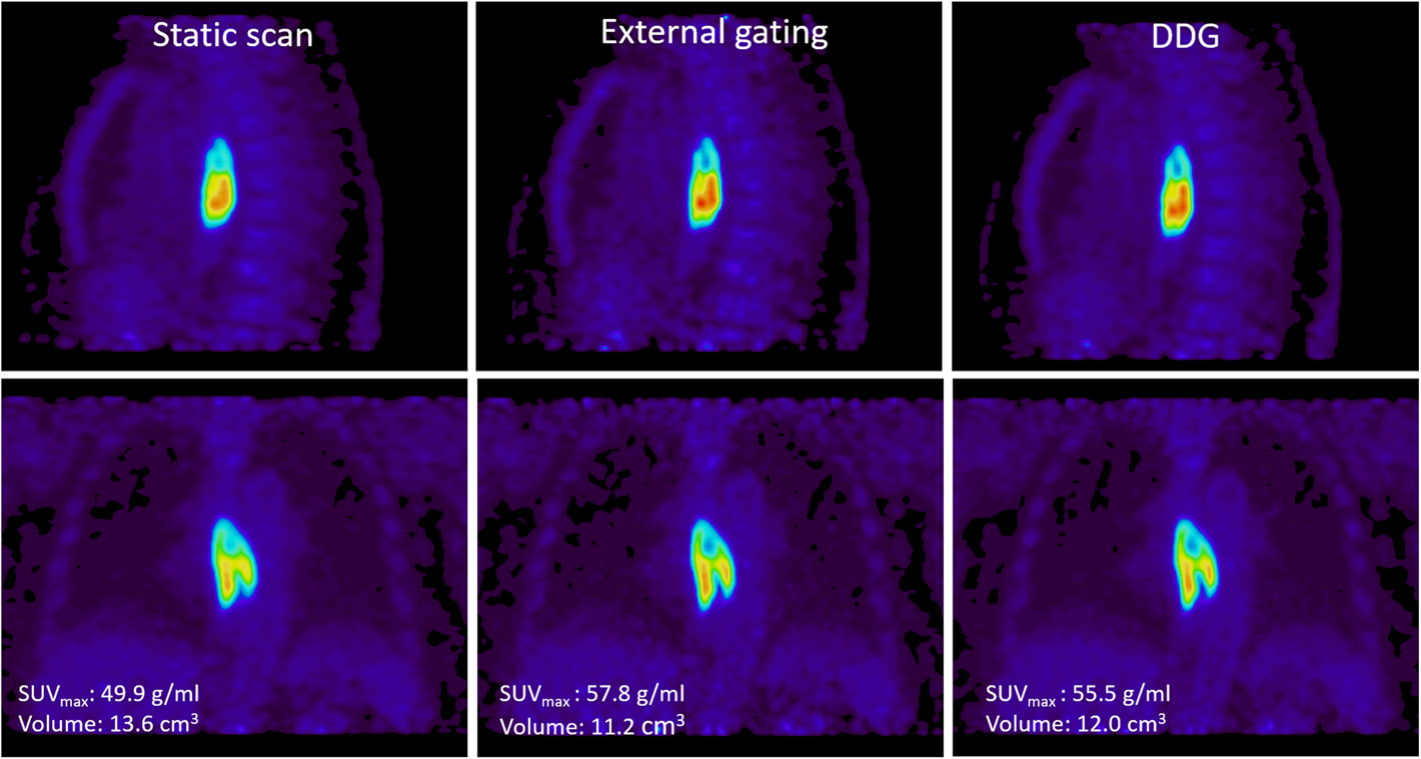
Example of a patient with an esophageal tumor. Images reconstructed from static raw data (left) and from raw data based on external hardware gating (middle) and DDG (right)

No difference in SUV_max_ or lesion volumes was found between images based on data-driven gating and images based on external device gating, but we found that the noise level in images based on data-driven gating was significantly lower (noise level 0.165) than in images based on either external hardware gating (noise level 0.235) or regular static acquisition (noise level 0.223 and 0.225). Reduced noise level implies higher signal-to-noise values (Table 1) and thus improved image quality for the DDG images in this study, which is also visible in Fig. 4. This result is in contradiction with Walker et al. (Walker et al. 2020) who however only visually investigated the noise level in the liver and bone marrow in PET/CT data and found almost equivalent noise levels in images based on DDG, external hardware gating, and a static image from 50% of the coincidence data. In another study, evaluating respiratory gating based on signals extracted from PET raw data using PCA, the results showed significantly lower noise levels in images based on respiratory gating signals extracted from PET raw data using PCA compared with gated images based on respiratory gating signals extracted from external bellow device (Fürst et al. 2015).

In order to evaluate and put the image quality improvement that respiratory gating achieves, in relation to the normal variation in image quality of two static acquisitions, we performed a test-retest study of the whole 6-min scan as two 3-min static acquisitions, the first 3 min and the last 3 min of acquisition. Interestingly, the paired *t*-test demonstrated a significant difference in SUV_max_ between the two static acquisitions. But still, both demonstrated lower average SUV_max_ than the DDG and external hardware gating provide. No significant difference in lesion volume was found between the two static acquisitions. Furthermore, the increase in SUV_max_ and decrease in lesion volume that breathing gating produces (see Fig. 3) appear to be large in relation to the scan-rescan variation of two static acquisitions.

**Fig. 5:**
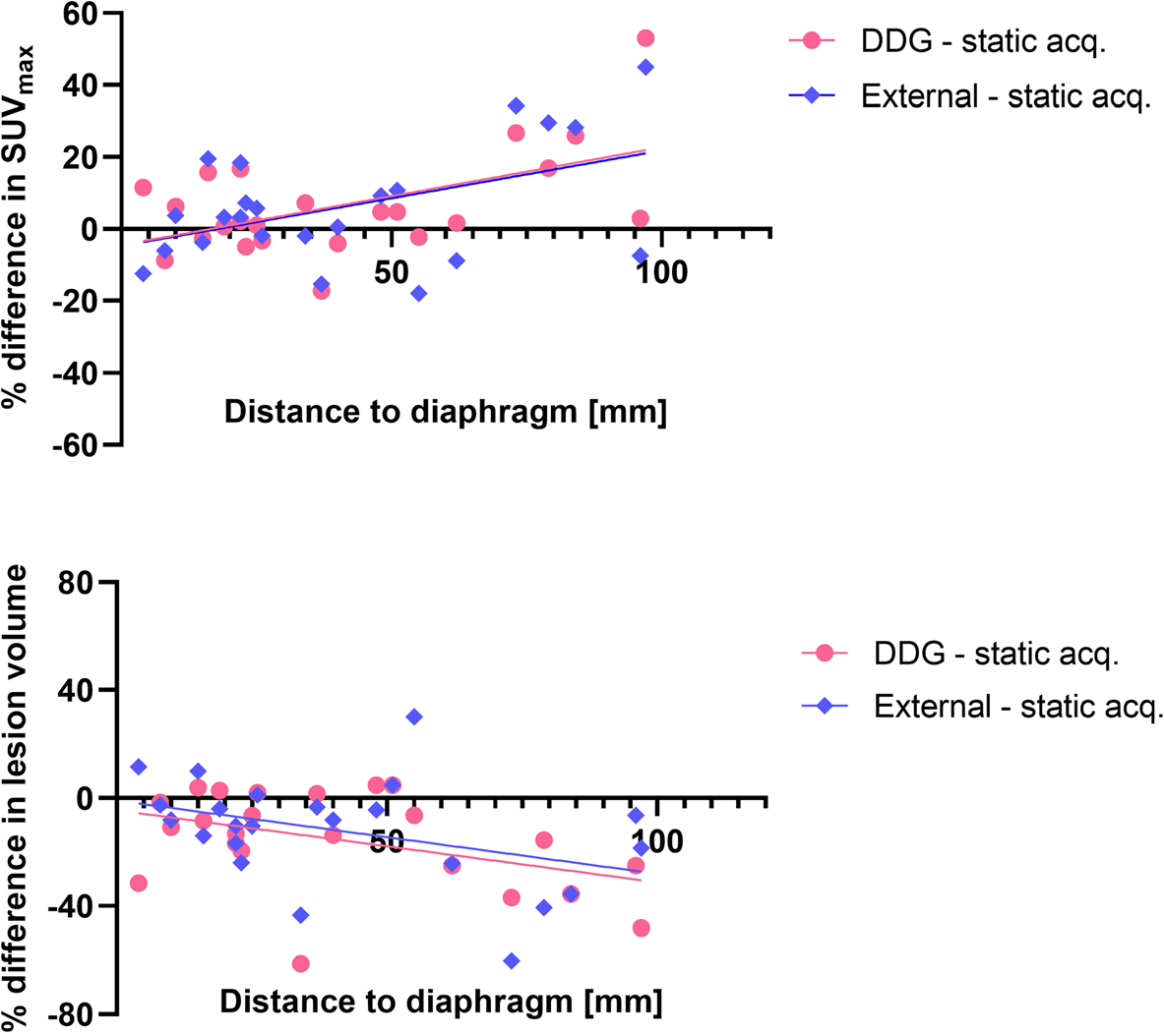
Relation between a lesion’s distance to the diaphragm and the improvement in image quality (upper graph: difference in SUV_max_; lower graph: difference in lesion volume). A week trend in the data can be seen, but the *R*^2^ < 0.3 and is not significant

**Fig. 6:**
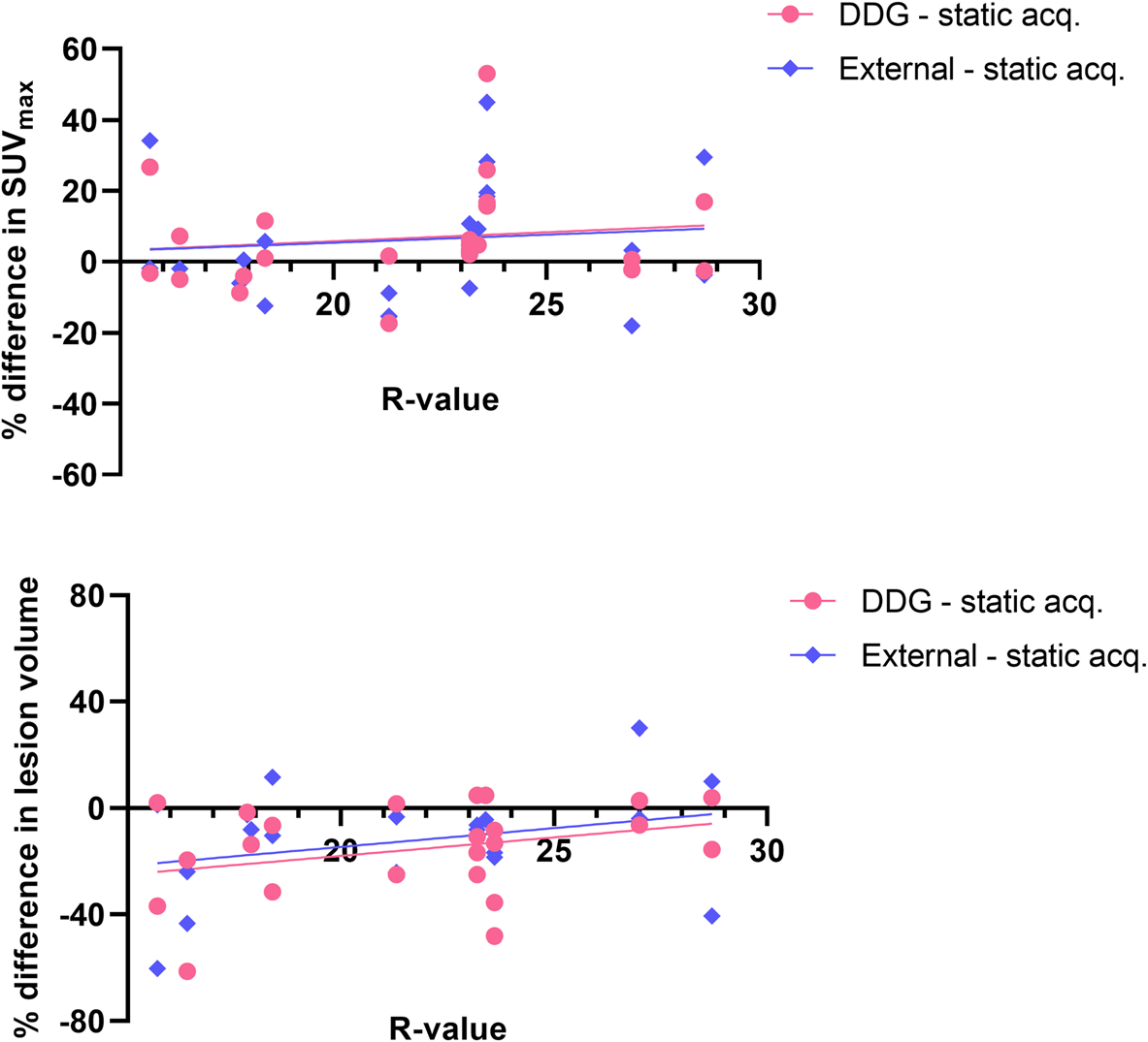
Correlation between DDG *R* value and difference in SUV_max_ (upper) respectively difference in lesion volume (lower). A week trend in the data can be seen, but the *R*^2^ < 0.2 and is not significant

The range of SUV_max_ values presented in this study was larger for the gating methods, both DDG and external hardware gating, than for the static acquisitions. The results demonstrated that breathing gating had larger impact on some patients or lesions than on other, but the increase in SUV did not have a strong correlation with an increased *R*-value or the lesion's distance to the diaphragm, see Figs. 5 and 6. Overall, the agreement in SUV_max_ between DDG and external device gating was very good in this study. In small lesions, < 1 cm^3^, the uncertainty in quantitative measurements becomes larger, and it is thus also in these lesions that we found the largest discrepancies between the gating methods (illustrated in Figs. 1 and 2).

We hypothesized that the impact from breathing gating methods would be larger in lesions located close to the diaphragm than more peripheral, but the results did not show any correlation between the lesion’s distance to the diaphragm and increase in SUV_max_, either between DDG and static acquisition or between external gating and static acquisition. In other studies, it seems like this parameter is of importance, at least when determining the *R* value or the impact of breathing motion in relation to location of the bed position, where PET beds located close to the diaphragm suffered from larger impact of motion (Beyer et al. 2003). In this study, a single bed position is included, and our results suggests that the location within that bed position is not relevant for the effect of breathing gating.

The present study shows a similar performance of DDG and external device gating in patients with esophageal tumors in a PET/MRI scanner. However, data-driven gating has some additional benefits compared to external device gating. First, and most important, it can be applied without need for any extra external device, which is time saving during the scanning procedure. Second, DDG resulted in reduced image noise compared to external device gating and static images. Third, it removes the risk of a possible time-synchronization error that can be introduced if the timing between the external device and the scanner is badly calibrated. Fourth, DDG can be used retrospectively in patients where image blurring has occurred due to extensive respiratory motion, but in that case at the expense of losing approximately 50% of the coincidence data.

### Limitations

The results and analysis in this study are focused on quantitative measures, such as SUV_max_, lesion volumes, and noise. Images have been visually assessed with respect to detect image artifacts and reconstruction errors, but the study does not include any thorough visual evaluation by an experienced observer. The relatively small number of patients and lesions is a limitation of this study, but despite this, clearly significant results were found.

## Conclusion

Data-driven gating with MotionFree for PET-MRI performs similar to external device gating for esophageal lesions. Both gating methods significantly increased the SUV_max_ and reduced the lesion volume in comparison with non-gated static acquisition. DDG resulted in reduced image noise compared to external device gating and static images.

## Data Availability

The datasets used and/or analyzed during the current study are available from the corresponding author on reasonable request.

## Abbreviations

DDG: Data-driven gating
FOV: Field of view
GEJ: Gastroesophageal junction
MRI: Magnetic resonance imaging
PET: Positron emission tomography
PCA: Principal component analysis
SNR: Signal-to-noise ratio
SUV: Standardized uptake value
